# Epidemiology of respiratory symptoms in children with Down syndrome: a nationwide prospective web-based parent-reported study

**DOI:** 10.1186/1471-2431-14-103

**Published:** 2014-04-15

**Authors:** Ruud HJ Verstegen, Roeland WNM van Hout, Esther de Vries

**Affiliations:** 1 Department of Pediatrics, Jeroen Bosch Hospital, PO Box 90153 ‘s-Hertogenbosch 5200 ME, The Netherlands; 2 Department of Pediatrics, Radboud University Medical Centre, Nijmegen, The Netherlands; 3 Department of Linguistics, Radboud University, Nijmegen, The Netherlands

**Keywords:** Down syndrome, Children, Respiratory symptoms, Epidemiology, Ear-nose-throat

## Abstract

**Background:**

Children with Down syndrome suffer from recurrent respiratory tract and ear-nose-throat complaints that influence daily life. Little is known about the frequency of these complaints, as well as their relation to co-morbidity and ageing.

**Methods/design:**

A prospective web-based parent-reported observational study was designed for parents having a child with Down syndrome (age 0 to 18 years). Upon registration, parents receive an email containing a link to a weekly questionnaire regarding respiratory symptoms during two consecutive years. Additionally, at the beginning, after one year and at the end of the study they receive an extended questionnaire concerning baseline data, daily activities and medical history. The data will be compared to the ongoing “child-is-ill” study, which collects weekly data in an identical fashion in children that are considered to be “normal as to being ill” by their parents.

**Discussion:**

This study will provide important data on the epidemiology of respiratory symptoms in children with Down syndrome, which will be useful for further studies on treatment options. Also, this study will gain insight in healthcare usage and work absence due to the child’s illnesses.

## Background

Down syndrome (DS) is the most common genetic cause of developmental delay in humans, affecting approximately 1 in 700 liveborn infants in the Netherlands [1,2]. Numerous health issues are related to DS. In newborns, hypotonia, facial characteristics and congenital heart disease (CHD) are variably present. Later in life, DS is associated with an increased incidence of hematological malignancies and auto-immune diseases, such as celiac disease, hypothyroidism and type 1 diabetes mellitus [3].

Respiratory complications and ear, nose and throat (ENT) diseases are a major contributor to morbidity and mortality in patients with DS. Up to 80% of all hospitalizations and admissions to a pediatric intensive care unit of children with DS is caused by lower respiratory tract infections [4,5]. The risk of respiratory syncytial virus infection and viral induced wheezing, but not asthma, is increased in infants with DS [6-11]. Moreover, up to 29% of deaths in DS are related to pneumonia, influenza and aspiration, with increased standardized mortality odds ratio of 3.00-4.15 in children with DS aged <20 years [12,13]. In addition, we recently showed that parent-reported recurrent respiratory tract infections in 8-year-old children with DS are associated with more impaired mental and motor development, lower health related quality of life and more behavioral problems compared to children whose parents report no increased respiratory tract infections in their child [14].

In a retrospective Finnish chart study, up to 40% of children and young adults (aged <30 years) with DS have had at least one pneumonia, partly caused by aspiration [15]. Schieve *et al.* report that 27.6% of children with DS compared to 17.5% of children without DS or other cause of mental retardation had symptoms of head/chest cold in the 2 weeks prior to the conduction of the survey [9]. Another study showed that 17.6% of school-aged children with DS have a continual runny nose and that 12% have had more than 3 upper respiratory tract infections in the preceding year [16]. ENT diseases are more frequently found in children with DS and include ear infections, hearing impairment and obstructive sleep apnea syndrome, [17-21] which can subsequently lead to behavioral problems and more developmental delay [22,23].

Treatment, and especially prevention of recurrent respiratory tract infections is challenging. Anatomical and physiological changes in the respiratory and ENT tract in children with DS are inherent to the syndrome and leave little room for improvement, except adenotomy and/or tonsillectomy. The use of prophylactic antibiotics to decrease the frequency of respiratory infections has never been studied in children with DS (nor in healthy children). Also, although palivizumab is sometimes prescribed in children with DS to prevent severe RSV disease, there have been no studies to evaluate the effect of this treatment. At last, other – more active – diagnostics and treatment by ENT specialists may be beneficial since adenotomy and/or tonsillectomy do not help all children [24]. In order to evaluate treatment options there is a need for more data on the exact incidence of respiratory symptoms and their evolvement in time to define patient groups who may benefit from these therapies. In this paper we describe the construction of a web-based observational study of respiratory tract infections in children with DS.

### Aim of the study

The primary goal of this study is to describe the frequency of respiratory symptoms in relation to age and comorbidity, with special focus on development of symptoms in time. The secondary goal is to show the medical and social consequences of respiratory symptoms such as doctor visits, absence from school and care leave of parents. Thirdly, we will relate the presence of co-morbidities in children with DS and their development in time with the obtained data.

### Methods/Design

We designed a prospective web-based parent-reported observational study for which inclusion started in March 2012. Approval of the study protocol was obtained from the Medical Ethical Review Board “METOPP”, Tilburg, the Netherlands.

#### Inclusion of participants

Parents or legal guardians of a child with DS in the Netherlands aged 0-18 years are eligible for inclusion in this study. By estimation, the total age cohort consists of approximately 4500 children. Specialized pediatric outpatient clinics for DS were contacted and received posters for their waiting room. Also, the Dutch Down Syndrome Foundation and related organizations made the study more widely known by publishing a call on their websites. Finally, social media were used to increase awareness of this study. Parents who are interested can obtain more information about and register for this study though the study website. According to Statistics Netherlands, almost all families with children in the Netherlands have access to the Internet and would therefore be eligible to participate [25]. However, parents should be able to understand Dutch in order to be capable to register for this study.

#### Data collection

All data for this study will be collected through web-based questionnaires. Participants receive email invitations, which are sent by an automatic data managing system (Research Manager, Nova Business Software, the Netherlands). At baseline, the parents are asked to complete a questionnaire, which includes questions on the composition of the household of the child, daily activities of their child (i.e. child care attendance or visiting primary or secondary school) and medical history of the child and family (Table 1). Thereafter, parents continue to receive a weekly questionnaire to ask whether their child has had symptoms in the past week. If so, additional questions, regarding the symptoms and consequences of the symptoms are asked (Table 2). After one year and at the end of the study (at two years) parents are asked to complete the baseline questionnaire again to determine any changes. Reminders will be sent for the baseline and two follow-up questionnaires twice.

**Table 1:** Annual questionnaire regarding background, daily activities and medical history of participating child with Down syndrome

**General questions**							
Child with Down syndrome		Date of birth					
		Gender					
Father/Mother		Date of birth					
History of allergy, asthma and/or eczema?		Yes		No			
Siblings		Number of older siblings					
		Number of younger siblings					
History of allergy, asthma and/or eczema?		Yes		No			
Does anyone smoke (almost) daily within the house?		Yes		No			
**Daily activities**							
*Divide the 14 half-days present in each week between the following activities:*		Home			Grandparents/family/host family		
		Child day care			Special needs day care		
		Playgroup (age 2-4y)			Pre-school kindergarten (age 4-5y)		
		Primary school (age 6-12y)			Special primary school		
		Secondary school			Special secondary school		
		Work placement			Working		
		Other					
If attending regular education, what grade is your child in?							
**Medical history**							
Compared to other children with the same age, the frequency of being ill is:		Lower		Equal	Higher		
*Does your child have a history of any of the following illnesses, complaints or medication usage?*							
Congenital heart disease		Yes		No			
If yes, please specify		VSD	ASD	AVSD	Tetralogy of Fallot	Other	Unknown
If yes, was surgery performed?		Yes		No			
Hypothyroid disease		Yes		No			
If yes, diagnosed at what age?							
Diabetes mellitus		Yes		No			
If yes, diagnosed at what age?							
Congenital malformations of the gastrointestinal tract		Yes		No			
If yes, please specify:		Oesophageal atresia		Duodenal atresia		Imperforate anus	
		Other		Unknown			
Celiac disease		Yes		No			
If yes, diagnosed at what age?							
Impaired hearing		Yes		No			
If yes, diagnosed at what age?							
Chronic snoring		Yes		No			
If yes, present since what age?							
Breathing with open mouth		Yes		No			
If yes, present since what age?							
Frequently suffering from serious colds		Yes		No, but did in the past			No
If complaints used to be present, until what age?							
Wheezing		Yes		No, but did in the past			No
If complaints used to be present, until what age?							
Eye disorders		Yes		No			
If yes, please specify:		Cataract	Glaucoma	Strabismus		Amblyopia	
		Wears glasses		Other		Unknown	
Leukaemia		Yes		No			
If yes, diagnosed at what age?							
Antibiotic use for respiratory tract/ENT* infections in the past year		0-5 times		6-10 times more than 10 times			
Hospital admission for RSV infection <2 years		Yes		No			
ENT-surgery		Yes		No			
If yes, please specify:		Tympanic tubes		Adenoidectomy			Tonsillectomy
Daily antibiotic prophylaxis		Yes		No, but did in the past			No
Inhaled corticoid for coughing, mucus and/or wheezing		Yes		No, but did in the past			No

*ENT Ear-nose-throat.

**Table 2:** Weekly questionnaire regarding medical symptoms in the past week

**Did your child have had any symptoms in the past week?**	**No**	**Yes**		
If yes,*				
Did you visit a doctor with your child?	No			Yes, general practitioner
	Yes, paediatrician			Yes, ENT-specialist^#^
	Yes, other doctor			
Did your child receive antibiotic treatment?	No	Yes		
Which symptoms were present?	Earache	Running ear		Sore throat
	Stuffy nose	Runny nose		Headache
	Hoarse voice	Coughing/mucus		Shortness of breath
Was the temperature higher than 38.5°C (fever)?	No	Yes		Did not take a temperature
Did your child stay at home from school?	No	Yes		Not applicable
Did your child stay at home from work placement?	No	Yes		Not applicable
Did your child stay at home from work?	No	Yes		Not applicable
Did you or your partner stay at home from work?	No	Yes		Not applicable

*The additional questions are only shown after the first question is answered “yes”.

^#^ENT Ear-nose-throat.

Reference data is collected through the ongoing “child-is-ill” study, which collects weekly data in an identical fashion in children that are considered to be “normal as to being ill” by their parents. For this study over 750 children, aged 2-18 years, are registered.

#### Statistics

Because this is an observational study, we did not perform a power analysis. However, we estimate that 2,5% of parents of a child with DS participates in this study. Given the potential sample size of approximately 4.500 children with DS aged <18 years in the Netherlands, we estimate to include approximately 110 children. This group size will allow us to study a larger cohort of children with DS longitudinally. This cohort is a small fraction of the total DS population, and the way parents were selected is not random, having the consequence that bias may show up. We will carefully address potential bias, in particular by comparing all our outcomes with research data available on children with DS. A second restriction is that the size of the cohort may limit the possibilities of subgroup analyses. A final restriction is the uncertainty about the amount of missing data, as increasing numbers of missing data reduce power. It is conceivable that specific differences between children with DS and controls cannot be detected.

First, baseline characteristics of the study population will be presented in a descriptive manner using percentages. Second, mixed model analysis will be performed at the end of the study. This model allows analyzing data from repeated measurements as well as proper handling of missing data. Also, effect of age as well as time (including seasonal effects) can be taken into account. Early termination of study participation by request of the parents or by >4 weeks missing data prior to the endpoint will be marked as lost to follow-up, and dealt with accordingly. Analysis will be performed using IBM SPSS Statistics.

#### Handling and storage of data

Data will be handled and stored according to legal and privacy guidelines. Parents will enter data concerning their child in a central web-based database. Security is guaranteed with login names, login codes and encrypted data transfer. The data of all subjects will be coded and this coding will not be retraceable to the individual patient. First, parents are not asked to enter the name of their child or exact address. Second, the email address of the parents will be blinded, stored encrypted and not accessible to the researchers of this study. Access to the database will be granted to the principal investigator and local investigator. Data will be stored for 15 years.

## Discussion

Although respiratory tract and ENT diseases are frequently encountered in the care for children with DS, little is known about their epidemiology. We have designed a prospective web-based study, which allows us to describe the incidence of respiratory symptoms, as well as their relation to comorbidity, age and season. This information can help to evaluate the effect of future treatment options.

To our knowledge this is the first prospective study in patients with DS on epidemiological data of respiratory symptoms. Earlier studies were often based on retrospective chart reviews, which excludes complaints for which no medical attention was sought by the parents [5,7,15]. Also, there have been many studies based on a single questionnaire where parents were asked if complaints were present in the past year(s), which leads to recall bias [9,11,16,21]. Other authors have therefore used both chart review and retrospective questionnaires to minimize the effect of recall bias [4,6]. Some studies are based on hospital admission/discharge records or mortality records only, and all outpatient symptoms are missed [8,12,13]. We expect our study to give more insight in respiratory symptoms in children with DS, which may help DS specialists to improve health care for this group.

## Abbreviations

DS: Down syndrome; ENT: Ear – nose – throat; RSV: Respiratory syncytial virus; RTI: Respiratory tract infection.

## Competing interests

The authors declare that they have no competing interest.

## Authors’ contributions

RV wrote the baseline questionnaire of the study and the draft of the manuscript, and will run the study. RvH designed the statistical protocol. EdV initiated the “child-is-ill” as well as this study, and participated in its design and coordination and helped to draft the manuscript, and will supervise the study. All authors read and approved the final manuscript.

## Pre-publication history

The pre-publication history for this paper can be accessed here:

http://www.biomedcentral.com/1471-2431/14/103/prepub
